# Hepatitis C Virus-Induced Exosomal MicroRNAs and Toll-Like Receptor 7 Polymorphism Regulate B-Cell Activating Factor

**DOI:** 10.1128/mBio.02764-21

**Published:** 2021-11-02

**Authors:** Tsai-Ling Liao, Yi-Ming Chen, Shie-Liang Hsieh, Kuo-Tung Tang, Der-Yuan Chen, Ying-Ying Yang, Hung-Jen Liu, Sheng-Shun Yang

**Affiliations:** a Department of Medical Research, Taichung Veterans General Hospital, Taichung, Taiwan; b Rong Hsing Research Center for Translational Medicine, National Chung Hsing University, Taichung, Taiwan; c Ph.D. Program in Translational Medicine, National Chung Hsing University, Taichung, Taiwan; d Division of Allergy, Immunology, and Rheumatology, Taichung Veterans General Hospital, Taichung, Taiwan; e Genomics Research Center, Academia Sinica, Taipei, Taiwan; f Faculty of Medicine, National Yang Ming University, Taipei, Taiwan; g Translational Medicine Laboratory, Rheumatology and Immunology Center, China Medical University Hospital, Taichung, Taiwan; h Division of Gastroenterology and Hepatology, Taipei Veterans General Hospital, Taipei, Taiwan; i Institute of Molecular Biology, National Chung Hsing University, Taichung, Taiwan; j The iEGG and Animal Biotechnology Center, National Chung Hsing University, Taichung, Taiwan; k Division of Gastroenterology and Hepatology, Department of Internal Medicine, Taichung Veterans General Hospital, Taichung, Taiwan; l School of Medicine, Chung Shan Medical University, Taichung, Taiwan; m Institute of Biomedical Sciences, National Chung Hsing University, Taichung, Taiwan; Johns Hopkins Bloomberg School of Public Health

**Keywords:** hepatitis C virus, mixed cryoglobulinemia, exosomal microRNA-122, B-cell activating factor, TLR7

## Abstract

There are large gaps in understanding the molecular machinery accounting for the association of hepatitis C virus (HCV) infection with autoimmunity. Mixed cryoglobulinemia (MC) is the most common HCV-associated extrahepatic manifestation, which is characterized by B-cell lymphoproliferation and autoantibody production. B-cell activating factor (BAFF) is a member of the tumor necrosis factor family and plays an important role in B-cell proliferation. We explored the roles of hepatocyte-derived exosomal microRNAs (exo-miRNAs) and BAFF in the extrahepatic diseases of HCV infection. The exo-miRNA profiles were explored using a next-generation sequencing approach, followed by quantitative reverse transcription-PCR validation. The Toll-like receptor 7 (TLR7) polymorphism were analyzed using quantitative PCR. The biological function of exo-miRNAs and TLR7 polymorphism in BAFF expression was evaluated by using immunoblotting and enzyme-linked immunosorbent assay. Significantly increased levels of BAFF, exosomes, and TLR7 were found in HCV patients, particularly in those with MC (*P < *0.005). HCV-infected hepatocyte-derived miR-122/let-7b/miR-206 upregulated BAFF expression in human macrophages through exosome transmission and TLR7 activation. Analysis of a TLR7 single-nucleotide polymorphism (rs3853839) revealed that G-allele carriers had increased TLR7 transcripts, resulting in more BAFF expression induced by hepatocyte-derived exo-miR-122, compared to those in C-allele carriers (*P < *0.005). We identified HCV-infected hepatocyte-derived GU-enriched miRNAs (e.g., miR-122/let-7b/miR-206) as a TLR7 ligand that could induce BAFF production in macrophages through exosome transmission. The polymorphism in TLR7 is associated with the BAFF levels induced by exo-miR-122. It may be a potential predisposing factor of MC syndrome development.

## INTRODUCTION

Hepatitis C virus (HCV) infection is a major health problem (1). In addition to liver damage, numerous HCV extrahepatic manifestations (HCV-EHMs) have been reported among patients with HCV infection (2–4). Mixed cryoglobulinemia (MC), the most common HCV-EHM, is a systemic vasculitis primarily mediated by immune complexes and characterized by nonneoplastic B-cell lymphoproliferation (5). It has been hypothesized that the pathology of MC may depend on the interaction between HCV and immune cells, resulting in B-cell activation and expansion (6). Approximately 50% of HCV patients exhibit a wide range of MC symptoms (7). The reasons why MC appears in only a portion of HCV patients are still unclear, but host genetics or viral factors are thought to be key determinants (8).

We observed increased CD19^+^ B-cell counts in HCV patients with MC syndrome compared to those without MC or healthy control (HC) subjects (*P < *0.05) (9). B-cell activating factor (BAFF) belongs to the tumor necrosis factor (TNF) family and is a key regulator of B-cell differentiation and survival. Immunoglobulin secretion and alterations in expression have been initially associated with different autoimmune disorders (10). Toubi et al. (11) observed that elevated BAFF levels were associated with HCV-related arthralgia and cryoglobulins, suggesting that BAFF may play a role in HCV-related autoimmunity.

MicroRNAs (miRNAs) are endogenous, noncoding RNAs that mediate mRNA cleavage, translational repression, or mRNA destabilization (12). It has been reported that extracellular miRNAs could be present in almost all biological fluids associated with Argonaute-2 carrier proteins to make them resistant to degradation (13). In addition, mature miRNAs can integrate into late endosomes, and then miRNA-containing exosomes are released and captured by recipient cells. One strand of the exosomal miRNA is integrated into carrier proteins, which contain the miRNA-induced silencing complex, and then interacts with the target mRNA (14, 15). Accumulating evidence implicates miRNAs in modulating the host immune response in HCV infection and suggests that they are shuttled by exosomes to exert biological functions (16–18). The role of miRNAs in HCV-EHMs, however, remains unclear. Although the target cell of HCV infection is the hepatocyte, the majority of BAFF is produced by macrophages. We hypothesized that exosomes released from hepatocytes may carry HCV-induced miRNAs to macrophages, resulting in a key mode of intercellular communication against HCV infection (e.g., BAFF upregulation). The aim of this study was to explore the molecular mechanisms of HCV-EHMs.

## RESULTS

### Increased BAFF levels in serum from patients with HCV-associated MC.

**FIG 1:**
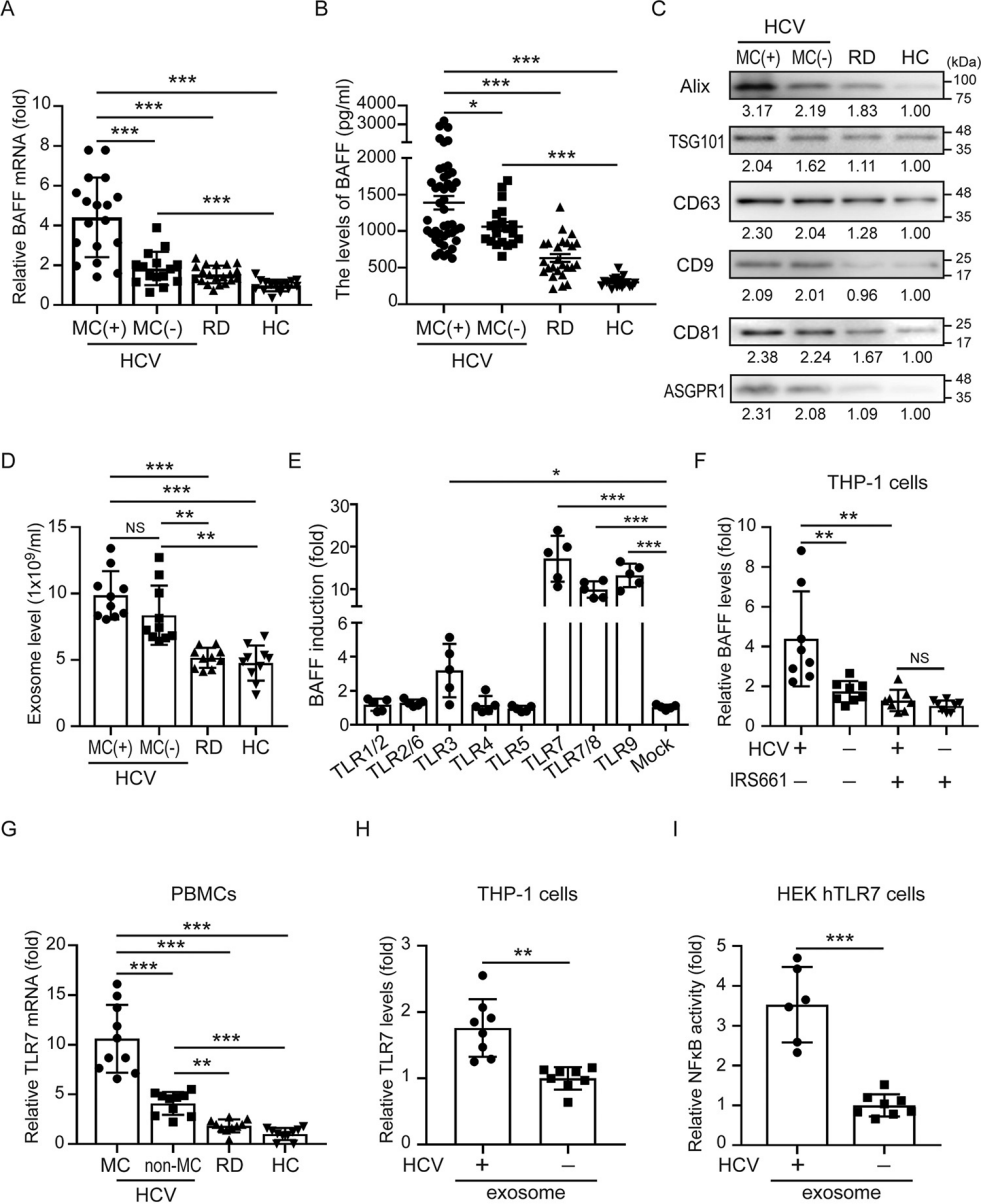
HCV patient-derived exosomes induced B-cell activating factor (BAFF) upregulation in macrophages through TLR7 activation. (A and B) Increased BAFF mRNA in PBMCs (A) and released levels in sera from patients with HCV-associated mixed cryoglobulinemia (MC) (B). (C) The expression of exosomal protein Alix (ALG-2-interacting protein X), TSG101 (Tumor Susceptibility Gene 101), CD63, CD9, CD81, and hepatocyte surface marker ASGPR1 (asialoglycoprotein receptor 1) in serum-derived exosomes was analyzed using immunoblotting. (D) Comparison of serum-derived exosomes levels in HCV patients with or without MC, rheumatic patients (RD), and healthy controls (HC). The serum-derived exosome levels in patients were quantified using ELISA-based assay. (E) Upregulated BAFF levels in PBMCs following TLR7/8/9 ligand stimulation. (F) Exosomes of HCV patient induced BAFF expression in THP-1 cell-derived macrophages, and this induction was suppressed in the presence of the specific TLR7 antagonist IRS661 (10 μM). (G) Increased TLR7 mRNA expression in PBMCs of patients with HCV-associated MC. (H) Exosomes of HCV patients induced TLR7 mRNA expression in THP-1 cell-derived macrophages. (I) HEK-Blue hTLR7 cells were stimulated with the exosomes of HCV patients or HC subjects for 24 h. NF-κB activation was evaluated in terms of the fold luciferase activity over control cells. All experiments were performed in triplicate, and data are presented as means ± the SD. *, *P < *0.05; **, *P < *0.01; ***, *P < *0.005.

We measured BAFF mRNA in PBMCs (Fig. 1A) and secretory levels in the sera (Fig. 1B) of patients with HCV infection using quantitative reverse transcription-PCR (QRT-PCR) and enzyme-linked immunosorbent assay (ELISA), respectively. BAFF levels were increased in the sera of patients with HCV-associated MC (*n* = 45, 1,389.0 ± 92.2 pg/ml) compared to those who had HCV infection but no MC (*n* = 20, 1,061.8 ± 61.6 pg/ml, *P < *0.05), those without infection (*n* = 25, 632.2 ± 53.9 pg/ml, *P < *0.005), or HC subjects (*n* = 20, 300.0 ± 14.8 pg/ml, *P < *0.005).

### Increased circulating exosomes in patients with HCV infection.

Exosomes are extracellular vesicles that play a key role in intercellular communication to disseminate pathogens or host-derived factors during infection (19). We hypothesized that hepatocyte-derived exosomes may play a key role in the regulation of BAFF expression in macrophages during HCV infection. To validate the purity of exosomes used in this study, we measured the particle sizes of extracellular vesicles (Zetasizer Nano ZS90; Malvern Instruments, Malvern, UK) and detected exosome-specific (Alix, TSG101, CD63, CD9, and CD81) surface markers using immunoblotting. We obtained extracellular vesicles with an average particle size of 134.9 nm, consistent with the typical exosomal size range of 50 to 150 nm (see Fig. S1 in the supplemental material). Moreover, these exosomes expressed specific exosomal markers (Fig. 1C). These results indicate that the serum-derived extracellular vesicles we extracted were composed mainly of exosomes. Moreover, most exosomes of HCV patients expressed hepatocyte-specific surface marker (asialoglycoprotein receptor 1 [ASGPR1]), suggesting their hepatic origin.

10.1128/mBio.02764-21.3 FIG S1 The proportion and particle size distribution of extracellular vesicles from the sera of HCV patients were measured by using nanoparticle tracking assay. Download FIG S1, EPS file, 2.7 MB. Copyright © 2021 Liao et al. 2021 Liao et al. https://creativecommons.org/licenses/by/4.0/ This content is distributed under the terms of the Creative Commons Attribution 4.0 International license.

To explore whether the exosome is a key factor in the regulation of BAFF expression during HCV infection, we analyzed and quantified the levels of exosomes in the sera of patients with HCV infection by using immunoblotting and ELISA-based assay. We observed that increased exosomes were detected in the sera of patients with HCV infection compared to those without infection (9.9 ± 1.8 × 10^9^ versus 4.8 ± 1.3 × 10^9^ exosome particles/ml, *P < *0.005; Fig. 1D). Among patients with HCV infection, there was no significance difference in exosome numbers between patients with and without MC symptom (9.9 ± 1.8 × 10^9^ versus 8.4 ± 2.2 × 10^9^ exosome particles/ml).

### Upregulated BAFF levels in PBMCs following TLR7 ligand stimulation.

We investigated whether innate immunity-associated Toll-like receptors (TLRs) might regulate BAFF expression in patients with HCV infection. Significantly increased BAFF levels in human peripheral blood mononuclear cells (PBMCs; *n* = 5) after TLR3 ligand poly(I·C) (3.19 ± 1.57-fold, *P < *0.05), TLR7 ligand imiquimod (17.18 ± 5.39-fold, *P < *0.005), or TLR7/8 ligand resiquimod (9.95 ± 1.93-fold, *P < *0.005) treatment suggest that double- or single-stranded RNA (e.g., miRNAs and viral RNA) might contribute to BAFF upregulation (Fig. 1E). In addition, the TLR9 ligand CpG also induced BAFF expression (13.28 ± 2.77-fold, *P < *0.005). Given that the aim of this study was to explore the role of miRNAs in BAFF upregulation and that TLR7 activation could induce the highest BAFF expression, we focused on the role of TLR7 in BAFF upregulation during HCV infection.

### HCV patient-derived exosomes induced BAFF upregulation in macrophages through TLR7 activation.

Next, we investigated whether increased circulating exosomes and TLR7 expression are associated with upregulated BAFF expression in patients with HCV infection. THP-1-derived macrophages were treated with exosomes from patients with or without HCV infection in the presence or absence of a specific TLR7 antagonist IRS661 (10 μM). After 24 h, intracellular BAFF expression levels were measured using QRT-PCR. The results revealed an increased level of BAFF expression in macrophages after the addition of exosomes from patients with HCV infection (*n* = 8, 4.53 ± 2.45-fold; Fig. 1F) compared to those without infection (*n* = 8, 1.80 ± 0.52-fold, *P < *0.01). This induction was suppressed by IRS661, suggesting that TLR7 is required for BAFF upregulation induced by HCV-infected patient-derived exosomes.

To further examine the association between TLR7 expression and HCV infection, we measured the levels of TLR7 mRNA in PBMCs from patients with or without HCV infection by using QRT-PCR. As shown in Fig. 1G, an increased level of TLR7 mRNA was revealed in the PBMCs of patients with HCV infection, particularly those with MC symptoms (10.6 ± 3.4-fold), compared to those without MC symptoms (4.1 ± 1.1-fold, *P < *0.005), rheumatic patients without HCV infection (1.8 ± 0.7-fold, *P < *0.005), and HC (1.0 ± 0.6-fold, *P < *0.005).

To assess whether HCV-infected patient-derived exosomes induced BAFF expression (Fig. 1F) through TLR7 activation, we measured TLR7 levels in macrophages after the addition of exosomes. Our results revealed elevated TLR7 expression in cells after treatment with exosomes from HCV patients compared to those from control subjects (*n* = 8, 1.76 ± 0.43-fold versus 1.00 ± 0.17-fold, *P < *0.01; Fig. 1H). Finally, we used HEK293 cells stably expressing human TLR7 and containing an NF-κB reporter gene (InvivoGen, USA) as a specific cell model to validate whether HCV patient-derived exosomes modulate NF-κB and BAFF expression upon TLR7 engagement. As shown in Fig. 1I, an increase in NF-κB activity was induced in cells treated with exosomes of HCV patients compared to those in control subjects (3.53 ± 0.95-fold versus 1.00 ± 0.28-fold, *P < *0.005). Based on our results, we hypothesized that circulating exosomes may carry single-stranded RNA to contribute to TLR7 activation and increase BAFF expression in the macrophages of patients with HCV infection, particularly in patients with MC symptoms.

### HCV-infected hepatocyte-derived exosomes induce BAFF expression in macrophages.

**FIG 2:**
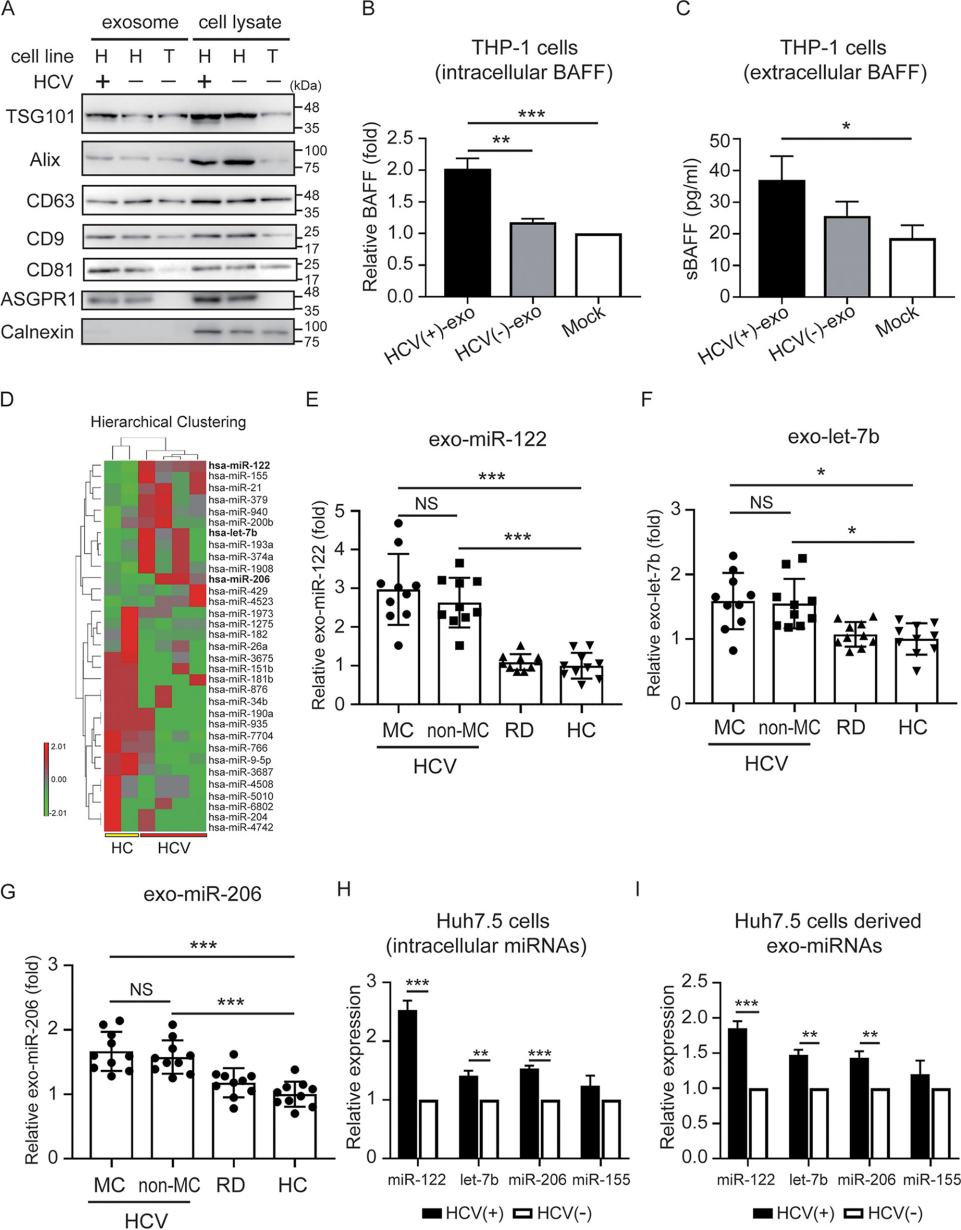
HCV-infected hepatocyte-derived exosomes carried microRNAs that induced BAFF expression in macrophage. (A) Expression of exosomal protein Alix, TSG101, CD63, CD9, CD81, and hepatocyte surface marker ASGPR1 in Huh7.5 cell-derived exosomes was analyzed using immunoblotting. The calnexin was used as negative control. H, Huh7.5 cells; T, THP-1 cells. (B and C) HCV-infected Huh7.5 cell-derived exosomes induced intracellular (B) and extracellular (C) BAFF expression in macrophages. (D) Hierarchical clustering of serum-derived exosomal microRNAs (miRNAs) profiles in HCV patients and healthy controls (HC) group using NGS analysis. The red color represents a relative expression greater than the median expression level across all samples, and the green color represents an expression level lower than the median. (E to G) Comparison of exo-miR-122 (E), exo-let-7b (F), and exo-miR-206 (G) levels in HCV patients with or without MC, rheumatic patients (RD), and HC. (H and I) Huh7.5 cells were infected with HCV at an MOI of 1 for 3 days, and the expression of intracellular (H) or exosomal (I) miRNAs was analyzed using QRT-PCR. All experiments were performed in triplicate, and data are presented as means ± the SD. *, *P < *0.05; **, *P < *0.01; ***, *P < *0.005.

Many types of cells secret exosomes into sera, and we further assessed the association between HCV-infected hepatocyte-derived exosomes and BAFF expression in macrophages by using an *in vitro* cell-based assay. Huh7.5 cells were infected with or without HCV for 3 days, and then exosomes were extracted from the supernatants. To validate that exosomes were of hepatocyte origin, we detected exosome (Alix, TSG101, CD63, CD9, and CD81)- and hepatocyte (ASGPR1)-specific surface markers using immunoblotting (Fig. 2A). The purified hepatocyte-derived exosomes were cocultured with THP-1-derived macrophages for 72 h, and the levels of intracellular and secretory BAFF expression were measured by using QRT-PCR and ELISA, respectively. A significantly elevated intracellular BAFF level was detected in THP-1-derived macrophages cocultured with exosomes from HCV-infected cells (2.02 ± 0.17-fold; Fig. 2B) compared to naive Huh7.5 cell-derived exosomes (1.18 ± 0.06-fold, *P < *0.01) and those without exosome treatment (1.00 ± 0.00-fold, *P < *0.005). Secretory BAFF revealed a similar trend (37.0 ± 7.6 versus 25.7 ± 4.5 versus 18.7 ± 4.0 pg/ml, *P < *0.05; Fig. 2C).

### Increased miR-122, let-7b, and miR-206 levels in HCV-infected hepatocytes and derived exosomes.

Our results showed that HCV-infected hepatocyte-derived exosomes could induce BAFF expression in macrophages. To explore the components carried by exosomes that could induce BAFF expression in macrophages, we compared the miRNA profiles in exosomes from the sera of HCV patients and HC subjects by using a next-generation sequencing (NGS) approach. After normalization, we observed 33 miRNAs that were distinctively expressed in exosomes from the sera of HCV patients compared to HC subjects (Fig. 2D; see also Table S1). Among the 13 upregulated exo-miRNAs, we focused on the top five candidates (miR-122, miR-155, let-7b, miR-21, and miR-206) and validated their expression by using QRT-PCR (Fig. 2E to G; see also Fig. S2). The results revealed increased levels of exo-miR-122 (2.97 ± 0.92-fold, *P < *0.005; Fig. 2E), exo-let-7b (1.59 ± 0.43-fold, *P < *0.05; Fig. 2F), and exo-miR-206 (1.67 ± 0.30-fold, *P < *0.005; Fig. 2G) in patients with HCV infection compared to rheumatic patients without infection or HC subjects. In addition, regardless of exo-miR-122, exo-let-7b, or exo-miR-206, there was no significant difference in their expression between HCV patients with or without MC symptoms.

10.1128/mBio.02764-21.2 TABLE S1 Differentially expressed miRNAs in serum-derived exosomes from patients with HCV infection compared to those in healthy controls (HC), identified by miRNA NGS analysis. Download Table S1, DOCX file, 0.02 MB. Copyright © 2021 Liao et al. 2021 Liao et al. https://creativecommons.org/licenses/by/4.0/ This content is distributed under the terms of the Creative Commons Attribution 4.0 International license.

10.1128/mBio.02764-21.4 FIG S2 Comparison of exo-miR-155 (A) and exo-miR-21 (B) levels in HCV patients with or without MC, rheumatic patients (RD), and HC. All experiments were performed in triplicate, and data are presented as means ± the SD. **, *P < *0.01; ***, *P < *0.005; NS, not significant. Download FIG S2, EPS file, 2.9 MB. Copyright © 2021 Liao et al. 2021 Liao et al. https://creativecommons.org/licenses/by/4.0/ This content is distributed under the terms of the Creative Commons Attribution 4.0 International license.

We further evaluated the association between the expression of these miRNAs and HCV infection by using an *in vitro* cell-based assay. Huh7.5 cells were infected with HCV at a multiplicity of infection (MOI) of 1 for 3 days, and the expression of intracellular or exosomal miRNAs was analyzed using QRT-PCR. The results indicated that miR-122 (2.53 ± 0.16-fold, *P < *0.005), let-7b (1.41 ± 0.08-fold, *P < *0.01), and miR-206 (1.53 ± 0.05-fold, *P < *0.005) levels were increased in Huh7.5 cells after HCV infection (Fig. 2H). In addition, elevated exo-miR-122 (1.85 ± 0.10-fold, *P < *0.005), exo-let-7b (1.47 ± 0.07-fold, *P < *0.01), and exo-miR-206 (1.43 ± 0.09-fold, *P < *0.01) levels were detected in Huh7.5 cell-derived exosomes after HCV infection (Fig. 2I). Despite increased miR-155 in the serum-derived exosomes of HCV patients (see Fig. S2), there was no significant difference in miR-155 expression in Huh7.5 cells or hepatocyte-derived exosomes after HCV infection, suggesting that increased exo-miR-155 in the sera of HCV patients may from originate from other cell types.

### miRNA-122/let-7b/miR-206 induced BAFF upregulation in macrophages through hepatocyte-derived exosome transmission.

**FIG 3:**
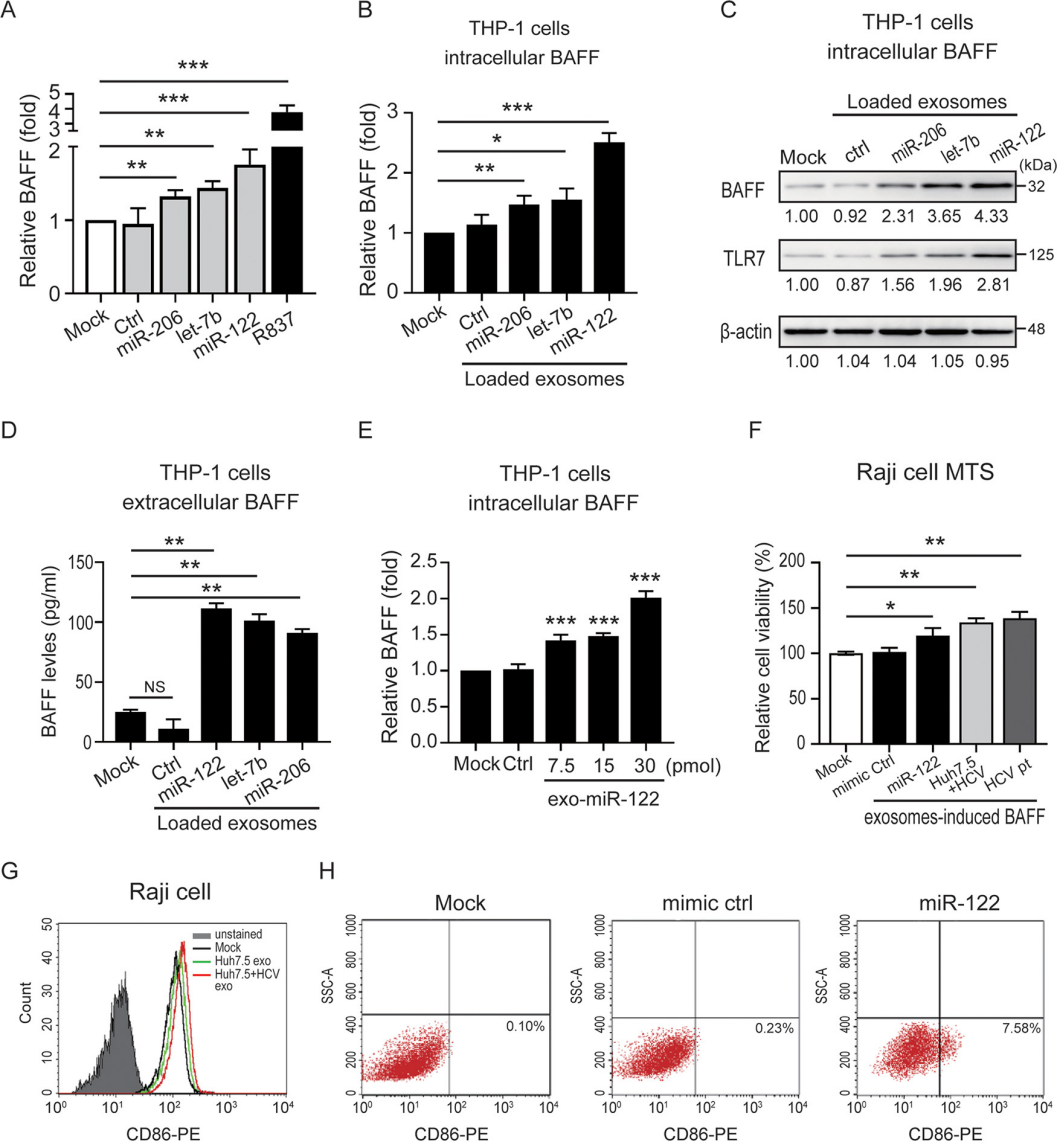
HCV-infected hepatocytes produced microRNA-induced BAFF upregulation in macrophages through exosome transmission. (A) miR-122, let-7b, and miR-206 mimic (10 μM) induced BAFF expression in normal human PBMCs. (B to D) miR-122, let-7b, or miR-206 in hepatocyte-derived exosomes contributes to BAFF expression in macrophages. Huh7.5 cell-derived exosomes were loaded with miR-122/let-7b/miR-206 mimic (10 μM) or control. THP-1-derived macrophages were cocultured with the indicated loaded exosomes for 24 h, respectively. The levels of intracellular BAFF mRNA (B), intracellular BAFF (C), and secretory BAFF (D) in THP-1 cells were measured using QRT-PCR, immunoblotting, and ELISA, respectively. (E) The hepatocyte-derived exo-miR-122 could induce BAFF expression in a dose-dependent manner. (F to H) HCV-associated exosomes induced-BAFF could stimulate B cell activation. Raji cells treated with HCV-associated exosomes (including HCV patient-derived exosomes, HCV-infected Huh7.5 cell-derived exosomes, and miR-122 mimic-loaded exosomes) induced BAFF for 96 h (F), and the cell viability (G) and the levels of CD86 (H) were measured using a cell proliferation assay and flow cytometry, respectively. All experiments were performed in triplicate, and data are presented as means ± the SD. *, *P < *0.05; **, *P < *0.01; ***, *P < *0.005.

Subsequently, we examined whether hepatocyte-derived miR-122, let-7b, or miR-206 could induce BAFF expression in macrophages. Human PBMCs were cocultured with the indicated miRNA mimics (10 μg/ml) for 24 h. The level of BAFF expression was evaluated by QRT-PCR. The results indicated that BAFF expression was significantly elevated after the addition of miR-122 mimic (1.76 ± 0.21-fold, *P < *0.005; Fig. 3A), let-7b mimic (1.44 ± 0.09-fold, *P < *0.01), or miR-206 mimic (1.32 ± 0.09-fold, *P < *0.01) compared to the mimic control (0.95 ± 0.22-fold) or no treatment (mock, 1.00 ± 0.00-fold).

Given that HCV infection could induce the levels of miR-122, let-7b, and miR-206 in hepatocyte-derived exosomes (Fig. 2I), and HCV-infected hepatocyte-derived exosomes could induce BAFF expression in macrophages (Fig. 2B and C), we further examined whether hepatocyte-produced miR-122, let-7b, and miR-206 could induce BAFF upregulation in macrophages through exosome transmission. MiR-122, let-7b, miR-206, or control mimics were loaded into Huh7.5 cell-derived exosomes by using electroporation in accordance with our previously published report (18). The levels of encapsulated miR-122/let-7b/miR-206 were measured using QRT-PCR (see Fig. S3A). Increased expression of individual miRNAs (miR-122, 1.2 ± 0.7 × 10^5^-fold; let-7b, 1.9 ± 0.1 × 10^5^-fold; and miR-206, 1.3 ± 0.3 × 10^5^-fold) was observed in Huh7.5 cell-derived exosomes after electroporation with miR-122/let-7b/miR-206 mimic, indicating its effective loading by electroporation. The exosomes were then added to THP-1-derived macrophages. After 72 h, significantly increased intracellular miR-122/let-7b/miR-206 levels (see Fig. S3B), accompanied by elevated intracellular BAFF expression (Fig. 3B and C) and secretory BAFF release (Fig. 3D), were observed in THP-1 cells with the addition of miR-122, let-7b, or miR-206 mimic-loaded exosomes. A higher BAFF level was induced in cells with miR-122 mimic-loaded exosomes treatment compared to let-7b or miR-206 (2.51 ± 0.16 versus 1.56 ± 0.18 versus 1.47 ± 0.15 -fold; Fig. 3B). To dissect whether the levels of exo-miR-122 are associated with BAFF expression in macrophages, THP-1-derived macrophages were treated with different concentrations of miR-122-loaded exosomes for 24 h, and the levels of BAFF were measured by using QRT-PCR. The results revealed that hepatocyte-derived exo-miR-122 could induce BAFF expression in a dose-dependent manner (*P < *0.005; Fig. 3E).

10.1128/mBio.02764-21.5 FIG S3 miR-122, let-7b, or miR-206 in hepatocyte-derived exosomes contribute to BAFF expression in macrophages. Huh7.5 cell-derived exosomes were loaded with miR-122/let-7b/miR-206 mimic or control. (A) The levels of miR-122, let-7b, or miR-206 loaded in exosomes were analyzed by QRT-PCR. THP-1-derived macrophages were cocultured with the indicated loaded exosomes for 24 h, respectively. (B) The levels of miR-122, let-7b, or miR-206 in THP-1 cells were measured using QRT-PCR. All experiments were performed in triplicate, and data are presented as means ± the SD. *, *P < *0.05; **, *P < *0.01; ***, *P < *0.005. Download FIG S3, EPS file, 2.6 MB. Copyright © 2021 Liao et al. 2021 Liao et al. https://creativecommons.org/licenses/by/4.0/ This content is distributed under the terms of the Creative Commons Attribution 4.0 International license.

### Hepatocyte-derived exo-miR-122 induced-BAFF could stimulate B cell activation.

Next, we examined the effect of HCV-associated exosome-induced BAFF on B-cell proliferation and activation. THP-1 cells were treated with exosomes from the sera of HCV patients or from the supernatants of HCV-infected Huh7.5 cells for 24 h. Secretory BAFF was collected and added to Raji cells for the cell proliferation assay. As shown in Fig. 3F, increased cell viability was detected in Raji cells following addition of HCV-associated exosome-induced BAFF (HCV patient-derived exosome, 138.7 ± 7.1%, *P < *0.01; HCV-infected Huh7.5 cell-derived exosome, 134.0 ± 4.6%, *P < *0.01). To further demonstrate whether BAFF induced by hepatocyte-derived exo-miR-122 had any effect on B-cell proliferation, Raji cells were treated with miR-122 mimic-loaded exosome-induced BAFF, and cell viability was measured by using the cell proliferation assay (Fig. 3F). Increased cell viability was observed in Raji cells treated with miR-122-loaded exosome-induced BAFF (119.7 ± 8.1%, *P < *0.05) compared to mimic control-loaded (101.7 ± 4.5%, *P < *0.05) or control cells (100.2 ± 1.8%, *P < *0.05). In addition, we detected elevated expression of CD86 (B-cell activation marker) in Raji cells after treatment with HCV-infected cell-derived exosome-induced BAFF (Fig. 3G). Increased activation was also shown in B cells after treatment with miR-122-loaded exosome-induced BAFF, compared to mimic control-loaded or control cells, by using flow cytometry (7.58% versus 0.23% versus 0.10%; Fig. 3H).

### Hepatocyte-derived exosomal miRNAs induced BAFF upregulation through TLR7 and NF-κB activation.

**FIG 4:**
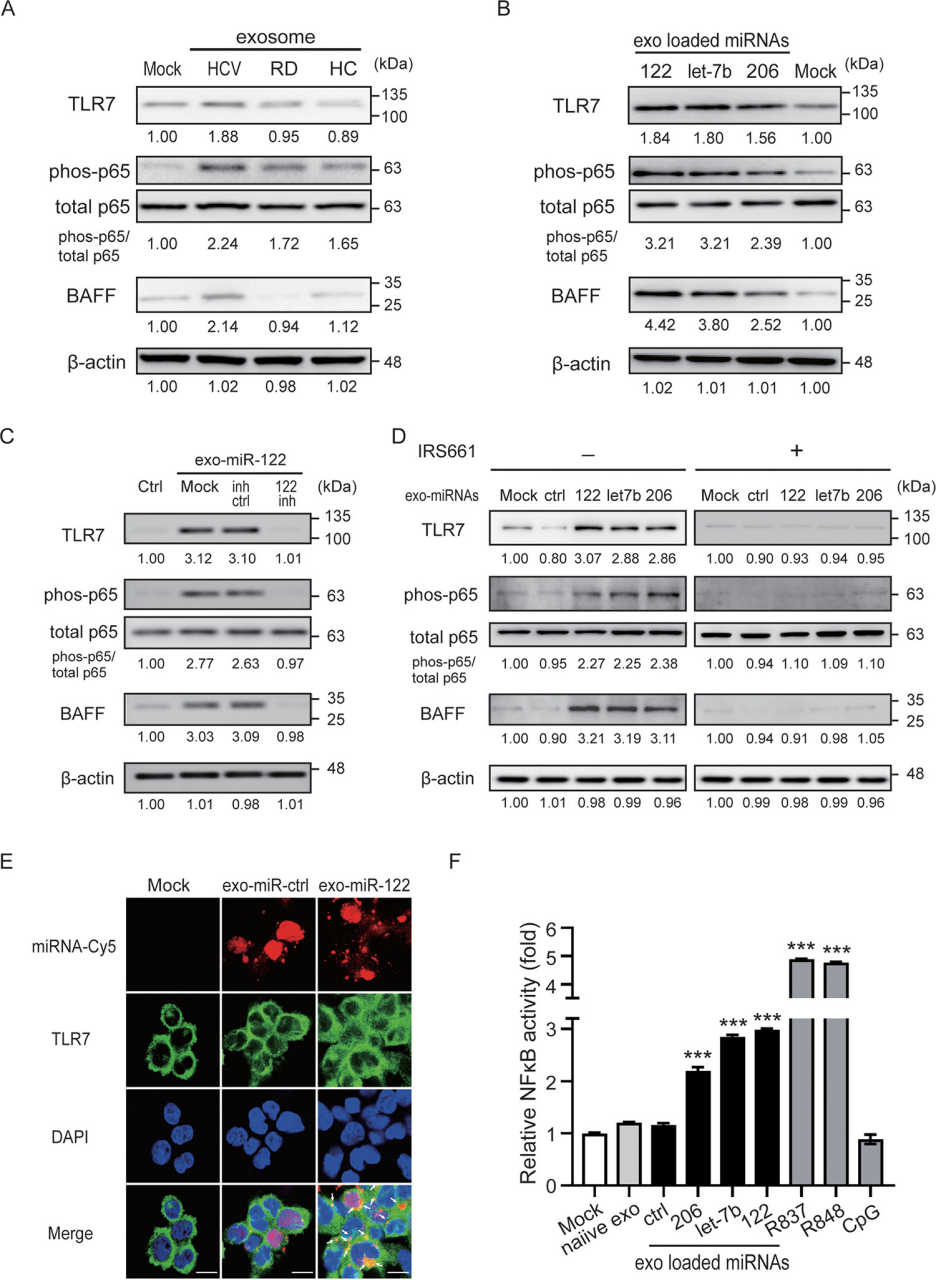
Hepatocyte-derived exosomal miRNAs induced BAFF upregulation through TLR7 and NF-κB activation. (A) Serum-derived exosomes of HCV patients induced TLR7 activation and increased phosphorylation of the p65 subunits of NF-κB in THP-1 cell-derived macrophages. (B) Huh7.5 cell-derived exosomes loaded with miR-122/let-7b/miR-206 induced TLR7 activation, accompanied by phosphorylation of p65 subunits and elevated BAFF levels. (C) Exosomes loaded with miR-122 mimic (10 μM) induced TLR7 activation in THP-1 cell-derived macrophages, and this induction was suppressed in the presence of miR-122 inhibitor (10 μM). (D) Exosomes loaded with miR-122/let-7b/miR-206 induced TLR7 activation in THP-1 cell-derived macrophages, and this induction was suppressed in the presence of specific TLR7 antagonist IRS661 (10 μM). (E) Huh7.5 cell-derived exosomes were loaded with Cy5-labeled miR-122 mimic (red) or mimic control and then cocultured with THP-1-derived macrophages for 4 h. Endogenous TLR7 was stained with TLR7 antibody (green). The miR-122 transmission and endogenous TLR7 in THP-1 cells was detected by using an immunofluorescence assay. The scale bar in the image represents 10 μm. (F) HEK-Blue hTLR7 cells were stimulated with miR-122-, let-7b-, or miR-206-loaded exosomes for 24 h. NF-κB activation was evaluated in terms of the fold luciferase activity over unstimulated cells. All experiments were performed in triplicate, and data are presented as means ± the SD. ***, *P < *0.005.

We further dissected the regulatory mechanism of hepatocyte-derived exo-miRNA induced BAFF upregulation. In addition to TLR7 activation, our results revealed a significantly increased phosphorylation of the p65 subunits of NF-κB in cells after treatment with exosomes from HCV patients compared to those from control subjects (Fig. 4A). Consistent with this, Huh7.5 cell-derived exosomes loaded with miR-122/let-7b/miR-206 induced TLR7 activation, accompanied by phosphorylation of p65 subunits, resulting in elevated BAFF levels (Fig. 4B). The effects of miR-122/let-7b/miR-206 mimic-loaded exosomes on TLR7 activation, the phosphorylation of p65 subunits, and BAFF production were suppressed in the presence of miR-122/let-7b/miR-206 inhibitor (Fig. 4C; see also Fig. S4A and B) compared to those in inhibitor control cells.

10.1128/mBio.02764-21.6 FIG S4 The effects of let-7b (A)- and miR-206 (B)-loaded exosomes on TLR7 activation, the phosphorylation of p65 subunits, and BAFF production were suppressed in the presence with its inhibitor (10 μM) compared to those in inhibitor control cells. Download FIG S4, EPS file, 2.4 MB. Copyright © 2021 Liao et al. 2021 Liao et al. https://creativecommons.org/licenses/by/4.0/ This content is distributed under the terms of the Creative Commons Attribution 4.0 International license.

To validate whether TLR7 is a key factor for BAFF upregulation, THP-1-derived macrophages were treated with miR-122/let-7b/miR-206-loaded exosomes in the presence of IRS661 (10 μM) to block TLR7 activation. As shown in Fig. 4D, the activation of TLR7 was diminished in the presence of IRS661, resulting in suppression of p65 subunit phosphorylation and inhibition of BAFF upregulation.

Next, we examined whether hepatocyte-derived miR-122 could bind to TLR7 on macrophages through exosome transmission by using immunofluorescence assay. Huh7.5 cell-derived exosomes were loaded with Cy5-labeled miR-122 mimic or mimic control and then cocultured with THP-1-derived macrophages for 4 h. miR-122 transmission and endogenous TLR7 in THP-1 cells were detected by using an immunofluorescence assay. As shown in Fig. 4E, Cy5-labeled miR-122 could be detected in THP-1 cells, suggesting that hepatocyte-derived miRNAs could be transferred to macrophages through exosome transmission. Compared to the miRNA mimic control, the colocalization of miR-122 and TLR7 was detected in macrophages, suggesting that miR-122 could bind to TLR7 specifically.

Finally, we used the HEK-hTLR7 cell model to validate exo-miR-122/let-7b/miR-206 modulation on NF-κB and BAFF expression upon TLR7 engagement. As shown in Fig. 4F, increased NF-κB activity was induced in cells stimulated with miR-122/let-7b/miR-206 mimic-loaded exosomes compared to control cells (miR-122, 2.99 ± 0.03-fold, *P < *0.005; let-7b, 2.85 ± 0.04-fold, *P < *0.005; and miR-206, 2.20 ± 0.07-fold, *P < *0.005).

### Genetic variation in TLR7 associated with upregulation of BAFF.

**FIG 5:**
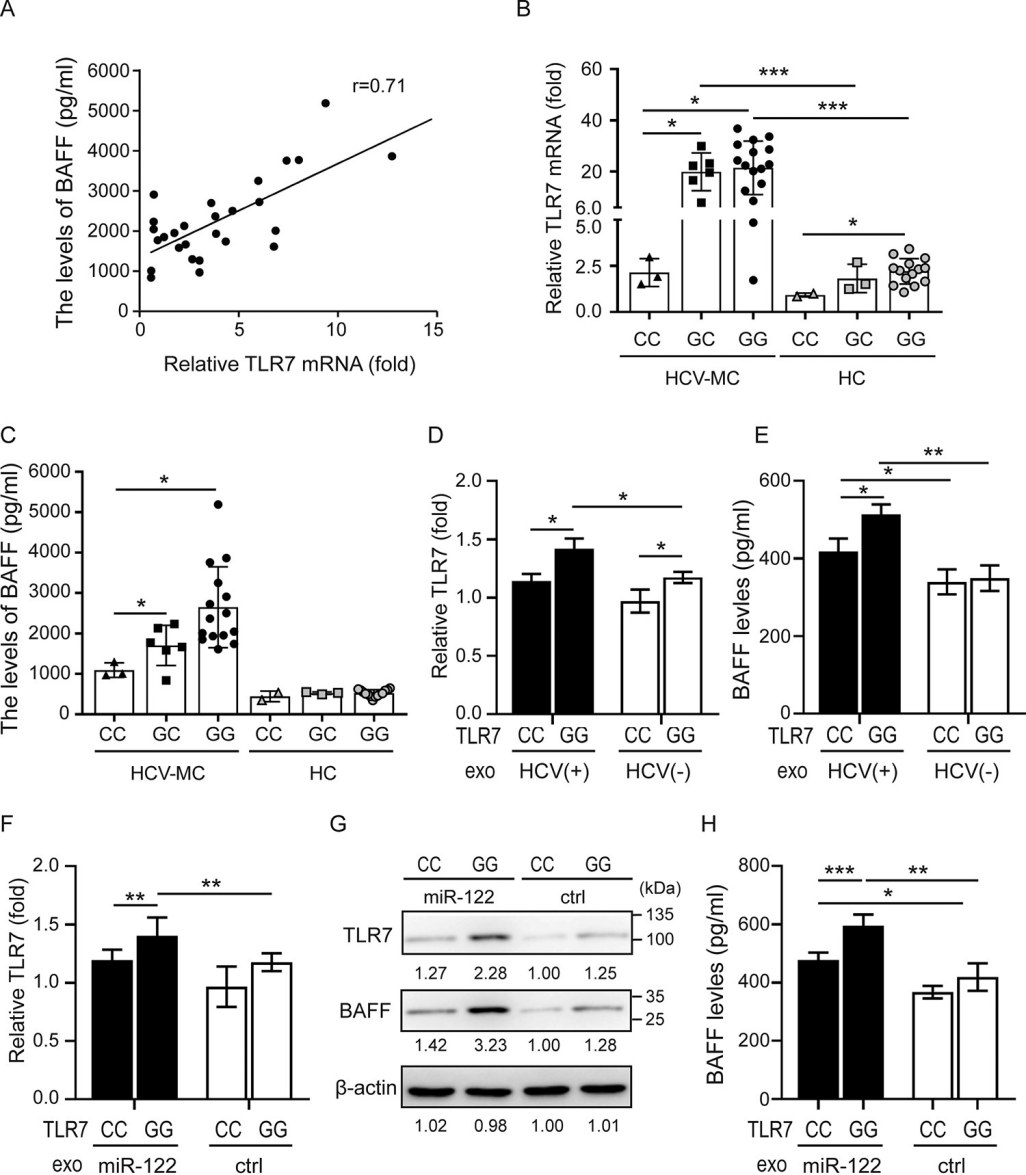
Genetic variation in TLR7 is associated with upregulation of BAFF. **(**A) The expression of TLR7 mRNA was positively correlated with BAFF release levels in patients with HCV-associated MC. (B and C) Correlation of the TLR7 transcript in PBMCs (B) and the secretory BAFF levels in the sera of HCV patients and healthy controls (HC) with rs3853839 TLR7 genotypes (C). Each symbol represents an individual, and horizontal lines indicate mean values. (D and E) Levels of TLR7 mRNA (D) and secretory BAFF expression (E) in the PBMCs of individuals with different rs3853839 TLR7 genotypes after treatment with HCV-infected hepatocyte-derived exosomes. (F to H) Levels of TLR7 mRNA (F), intracellular BAFF (G), and secretory BAFF (H) expression in the PBMCs of individuals with different rs3853839 TLR7 genotypes after treatment with miR-122-loaded exosomes. All experiments were performed in triplicate, and data are presented as means ± the SD. *, *P < *0.05; **, *P < *0.01; ***, *P < *0.005.

Accumulating evidence demonstrates that TLR7 expression is correlated with clinical outcomes in HCV infection (20–22). Given that we observed elevated BAFF levels (Fig. 1A) and higher TLR7 expression (Fig. 1G) in patients with HCV-associated MC compared to those had HCV infection but without MC, we analyzed the association between TLR7 levels and BAFF expression in HCV-associated MC patients (*n* = 27). As shown in Fig. 5A, we demonstrated a positive correlation between TLR7 expression levels and BAFF released levels in patients with HCV-associated MC (*r* = 0.71, *P < *0.005).

Shen et al. (23) identified that TLR7 SNP rs3853839 confers increased expression of TLR7 and showed that TLR7 transcription was increased in G-allele carriers but not C-allele carriers. To assess whether TLR7 SNP rs3853839 is a predisposing factor in HCV-associated MC, we analyzed the associations among TLR7 genotype, TLR7 mRNA expression, and released BAFF levels in patients with HCV-associated MC. In both HCV-associated MC patients and HC subjects, we observed that G-allele carriers had increased levels of the TLR7 transcript compared to C-allele carriers (HCV-associated MC, 21.45 ± 10.42 versus 2.14 ± 0.75-fold, *P < *0.005; HC, 2.21 ± 0.69 versus 0.93 ± 0.11-fold, *P < *0.05; Fig. 5B). Among HCV-associated MC patients, G-allele carriers had elevated BAFF release levels in comparison with C-allele carriers (2647.0 ± 258.2 pg/ml versus 1,093.2 ± 104.0 pg/ml, *P < *0.005; Fig. 5C). No significant difference in BAFF expression was found between G- and C-allele carriers in HC subjects, suggesting that elevated BAFF expression may be associated with HCV infection. Given that TLR7 is an X-linked gene that can undergo escape from X chromosome inactivation in immune cells (24), we further stratified the data in HCV-associated MC patients and analyzed differential TLR7 expression by sex (see Fig. S5). Among HCV-associated MC patients that carried G-bialleles, women displayed higher TLR7 transcriptional expression and BAFF secretion relative to men (TLR7, 26.81 ± 6.71-fold versus 18.18 ± 4.12-fold; BAFF, 2,715 ± 1,060 pg/ml versus 2,205 ± 226.9 pg/ml).

10.1128/mBio.02764-21.7 FIG S5 Correlation of the TLR7 transcript in PBMCs (A) and secretory BAFF levels in sera of HCV patients and healthy controls (HC) with rs3853839 TLR7 genotypes (B). G represents the major allele and C represents the minor allele of rs3853839. Each symbol represents an individual, and horizontal lines indicate mean values. All experiments were performed in triplicate, and data are presented as means ± the SD. *, *P < *0.05; ***, *P < *0.005. Download FIG S5, TIF file, 1.9 MB. Copyright © 2021 Liao et al. 2021 Liao et al. https://creativecommons.org/licenses/by/4.0/ This content is distributed under the terms of the Creative Commons Attribution 4.0 International license.

We showed that HCV induces exo-miR-122 production in hepatocytes, which upregulates BAFF expression in macrophages through TLR7 activation. To validate the effect of genetic variations in TLR7 on HCV-infected hepatocyte-derived exosomes/exo-miR-122 induced BAFF expression, HCV-infected Huh7.5 cell-derived exosomes (Fig. 5D and E) or Huh7.5 cell-derived exosomes loaded with miR-122 (Fig. 5F to H) were added to PBMCs from HC subjects with different TLR7 SNP genotypes (CC or GG). At 72 h posttreatment, BAFF levels were measured by using QRT-PCR, immunoblotting, and ELISA. As shown in Fig. 5D, slightly higher TLR7 mRNA expression was revealed in PBMCs of G-allele carriers compared to that in C-allele carriers (1.21 ± 0.05-fold versus 1.00 ± 0.10-fold, *P < *0.05). Increased TLR7 mRNA was induced after treatment with HCV-infected Huh7.5 cell-derived exosomes, especially in G-allele carriers (GG, 1.46 ± 0.09-fold; CC, 1.18 ± 0.06-fold, *P < *0.05). No significant difference was noted between BAFF levels in PBMCs of different SNP carriers after treatment of naive Huh7.5 cell-derived exosomes. After the addition of HCV-infected Huh7.5 cell-derived exosomes, elevated BAFF levels were detected, particularly in G-allele carriers (GG, 513.7 ± 25.5 pg/ml; CC, 418.1 ± 33.4 pg/ml, *P < *0.005). A similar trend was shown in treatment with miR-122-loaded exosomes (Fig. 5F to H).

## DISCUSSION

Accumulating evidence indicates an association between HCV-EHMs and B-cell abnormalities. Exosomes are vehicles for intercellular communication. It has been demonstrated that cellular/viral components could be carried by exosomes to provide communication between different cell types and regulate viral infection (19). Here, we observed an increased level of circulating exosomes in patients with HCV infection that was consistent with published literature (25, 26). A previous study detailed hepatocyte-derived exosomes containing HCV RNA that induce the production of IFN-α in plasmacytoid dendritic cells (pDCs) (27). Recently, several miRNAs have been identified as being involved in HCV infection (16–18); however, the role of HCV-associated miRNAs in B-cell disorders remains unclear.

In the present study, we observed elevated levels of exosome-carried miRNAs (e.g., exo-miR-122, exo-let-7b, exo-miR-206, and exo-miR-155) in the sera of patients with HCV infection. We further demonstrated that HCV-infected, hepatocyte-derived exo-miR-122/exo-let-7b/exo-miR-206 could upregulate BAFF expression in macrophages through TLR7 activation. Salvi et al. showed that human TLR7 is an miRNA receptor, but its activating capability is not shared by all miRNAs (28). Several studies described GU-rich elements, or the number of U ribonucleotides in the sequence composition, as important for innate immune activation by single-stranded RNAs (28–30). miR-122, let-7b, and miR-206 are GU enriched (see Fig. S6). MiR-122 is the most abundant liver-specific microRNA involved in various physiological processes in hepatic function (31) and is required for HCV replication (17). Let-7b has been demonstrated to play a negative role in HCV replication (32) and promote IFN-α secretion via TLR7 (28). Yang et al. demonstrated that miR-206 inhibited proliferation and invasion of Huh7 cells by targeting protein tyrosine phosphatase 1B (PTP1B) (33). Another study identified that PTP1B inhibitors robustly augmented the antiviral effects of IFN1 against HCV (34). Based on these published studies and our preliminary data, we proposed that miR-206 may be involved in HCV infection. However, further experimentation is required to elucidate the mechanism in detail.

10.1128/mBio.02764-21.8 FIG S6 Sequence of synthetic miR-122, let-7b, and miR-206. The GU-rich positions are underlined. Download FIG S6, EPS file, 2.1 MB. Copyright © 2021 Liao et al. 2021 Liao et al. https://creativecommons.org/licenses/by/4.0/ This content is distributed under the terms of the Creative Commons Attribution 4.0 International license.

Fabbri et al. demonstrated that specific miRNAs in tumor cell-released exosomes can reach and bind TLRs to induce a prometastatic inflammatory response (29). Recently, Wang et al. demonstrated that miR-122 released by injured liver elicits acute pulmonary inflammation via activation of the alveolar macrophage TLR7 signaling pathway, which was abolished by mutating the TLR7-binding sequence on miR-122 (35). We observed slightly elevated BAFF expression induced by exo-miR-122 compared to exo-let-7b or exo-miR-206, which may be associated with a higher binding affinity of miR-122 to TLR7. In addition, our *in vitro* result showed that hepatocyte-derived exo-miR-122 could induce BAFF expression in a dose-dependent manner. Higher levels of miR-122 were induced after HCV infection in comparison with let-7b and miR-206, which may contribute higher BAFF production. A study revealed that the severity of chronic hepatitis C and viral load correlated with miR-122 levels (36). We observed that HCV patients with viremia usually exhibited higher exo-miR-122 expression and elevated secretory BAFF levels, which may be associated with the host anti-HCV immune response. In the future, larger and more in-depth studies are required to confirm our observations and hypothesis.

In addition to liver damage, numerous extrahepatic manifestations have been reported among patients with HCV infection, which may be associated with deleterious B-cell proliferation (2–4). B cells have the unique ability to generate antibodies against pathogens. BAFF is a key factor in promoting proliferation and activation of B cells and has been identified as associated with human autoimmune disorders (10). Chronic infection may lead to increased release of BAFF and emergence of autoreactivity, especially in those with genetic susceptibility (37). A correlation between increased levels of BAFF and elevated B cell responses in hosts during pathogen invasion has been identified (38). Circulating auto-antibodies have been found in approximately half of patients with chronic HCV infection (39). Accumulating studies have reported elevated serum BAFF levels in HCV patients with lymphoproliferative disorders (11, 40); however, the mechanisms are still unclear. Our previous results revealed that about 81.8% (*n* = 45) of our rheumatic patients with MC syndrome had positive HCV IgG. An ∼1.55-fold increase in the CD19^+^ B-cell count was measured in patients with HCV-associated MC syndrome compared to those without MC syndrome or HC subjects (*P < *0.05) (9). In the present study, we observed increased levels of exosomes and exo-miR-122 in the sera of HCV patients or released from HCV-infected hepatocytes. We further demonstrated that HCV patient serum-derived, HCV-infected hepatocyte-derived, and miR-122-loaded exosomes could increase B-cell proliferation and activation through induction of BAFF upregulation in macrophages. Further in-depth studies are needed to confirm the induction of BAFF upregulation by HCV-associated exosomes and anti-HCV antibody overproduction by B cells to cause MC symptom development.

Previous reports revealed that the polymorphism −871C/T in the BAFF promoter was associated with the levels of BAFF mRNA in monocytes of patients with autoimmune diseases (41, 42). Gragnani et al. showed a significant association between MC phenotype and strong BAFF expression, demonstrating a significantly higher prevalence of T allele homozygosity in the group of patients with MC (8, 43). Consistent with previous studies, we observed a higher prevalence of the −871T genotype in HCV patients with MC compared to those without MC (28.6% versus 15%, *P < *0.01), suggesting that the genetic background of HCV patients may contribute to the development of extrahepatic manifestations.

TLRs play a role in the first line of defense during HCV infection. Our *in vitro* results showed increased BAFF expression in macrophages through TLR7/8/9 activation. A previous report showed that TLR9 activation induces the expression of BAFF and leads to increased proliferation of B cells in patients with SLE (44). A recent study demonstrated that TLR9 signaling triggers Th1 and rheumatoid factor autoantibody responses, suggesting that TLR9 plays a central role in the autoimmune response of HCV-MC (45). In addition to TLR9, TLR7 has been identified as a cellular factor associated with HCV replication (46). Accumulating evidence revealed that TLR7 expression and signaling are associated with outcomes in patients with HCV infection (20–22). Wang et al. suggested that the variation in TLR7 genes might impair immune responses during HCV infection (20). The TLR7 3′ UTR SNP rs3853839 (G/C) has been identified as strongly associated with TLR7 expression, which is thought to be a predisposing factor of SLE (23). Here, we observed significantly increased TLR7 mRNA expression in PBMCs of patients with HCV infection compared to those without infection (*P < *0.005). In addition, a positive correlation between TLR7 expression levels and BAFF-released levels was shown in patients with HCV infection (*r* = 0.71). Consistent with a published report, G-allele carriers of the TLR7 SNP rs3853839 showed increased TLR7 transcription compared to C-allele carriers, particularly in those with HCV infection. We noted higher BAFF expression in HCV patients who carried G-alleles with higher TLR7 levels, but there was no significant difference in BAFF expression in HC patients who were G- and C-allele carriers. Newman et al. (47) demonstrated that codon optimization of Tlr7 increases protein levels, as well as the response to ligands. Our results showed that HCV-induced BAFF upregulation through TLR7 activation and the levels of BAFF induced by HCV were positively correlated with TLR7 expression. The TLR7 SNP rs3853839 may be a predisposing factor in HCV-associated MC. Larger studies are needed to further confirm our observations.

To our knowledge, this is the first study to investigate the roles of exosomes and TLR7 genetics in HCV-associated autoimmune disease. Although we revealed some novel findings, the study has some limitations. First, it was conducted at a single medical center and included a small number of cases. Therefore, the study is not likely to reflect the complete characteristics of chronic HCV infection with extrahepatic manifestations. Second, the TLR7 gene is located on the human X chromosome and is thought to be sex-linked (48). Souyris et al. demonstrated that biallelic B lymphocytes from women displayed greater TLR7 transcriptional expression than monoallelic cells, correlated with higher TLR7 protein expression in female than in male leukocyte populations (24). In this study, we were unable to enroll equal numbers of both genders in both HCV and HC categories. Most patients were female, which may result in statistical bias. Therefore, we further stratified our data and analyzed differential TLR7 expression by sex (see Fig. S5). Our results revealed that patients who carried G-bialleles displayed higher TLR7 transcriptional expression than those with the monoallele. Moreover, women who carried G-bialleles displayed higher TLR7 transcriptional expression than did men. Our findings suggested that enhanced TLR7 expression owing to biallelism may contribute to increased BAFF expression, thus causing a higher risk of developing HCV-MC in women. This may explain why most HCV-MC patients are female in our study. Further larger cohort studies are needed to validate our hypothesis. Finally, this study was cross-sectional in design; thus, we cannot rule out the possibility that the immune response, exosome production, and exosomal miRNA expression changed due to the therapeutic strategies. However, we validated our clinical observations and hypothesis by using an *in vitro* assay, which still provides useful information.

**FIG 6:**
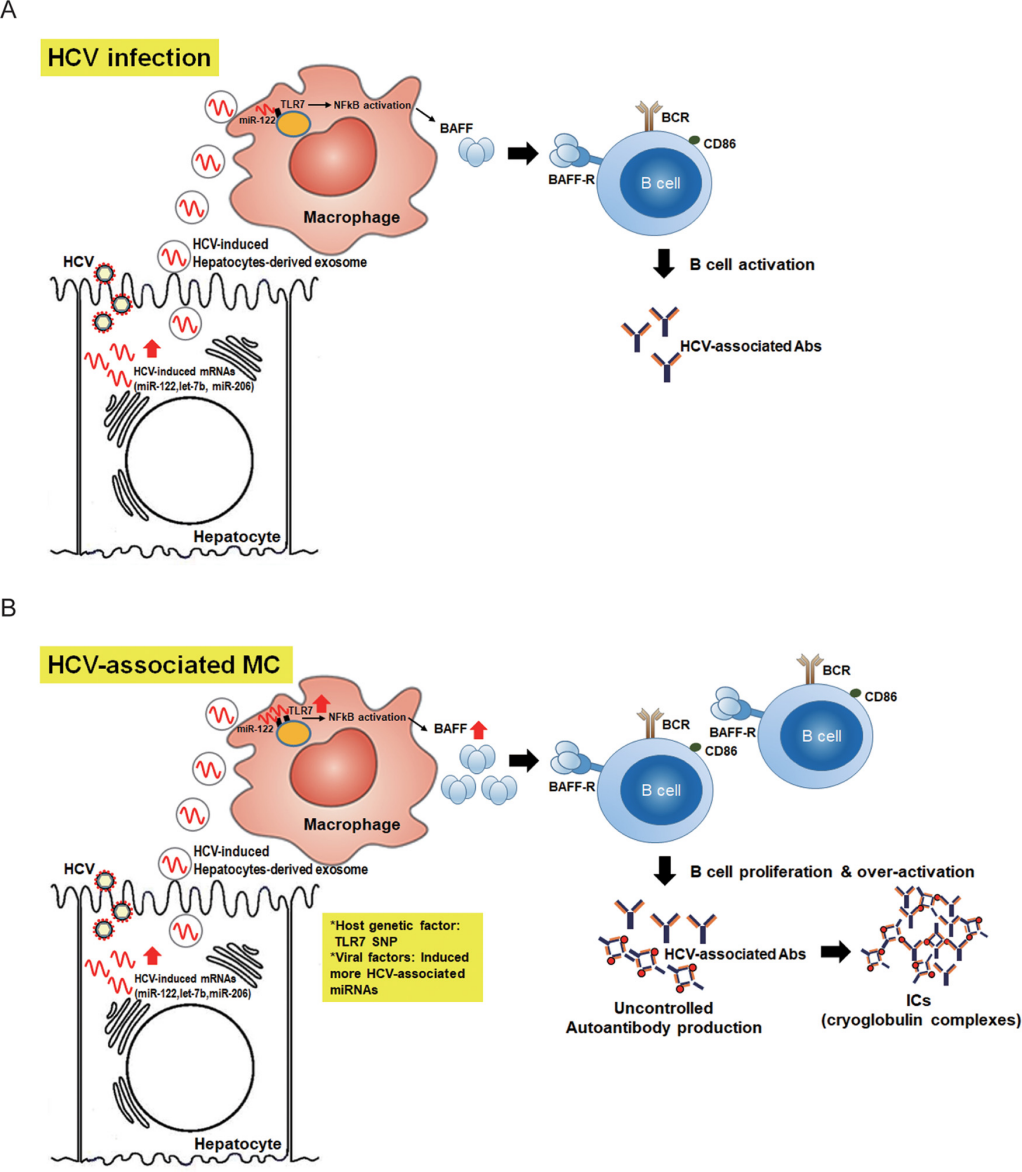
Proposed model for the biological role of hepatocyte-derived exo-miR122, TLR7, and BAFF in HCV infection (A) and HCV-associated MC development (B), based on the results of this study. (A) HCV induces elevated GU-enriched miRNAs (e.g., miR-122, let-7b, and miR-206) expression in hepatocytes during infection and then stimulates BAFF upregulation in macrophages through exosome transmission and TLR7 activation. HCV-associated exosomal miRNA-induced BAFF could stimulate B cell proliferation and activation and thus produce anti-HCV antibodies. (B) Hepatocyte-derived exo-miR-122-induced BAFF expression is a dose-dependent matter. In addition, G-alleles in TLR7 SNP (rs3853839) had an effect on increased TLR7 transcripts. miR-122 or TLR7 overexpression may induce BAFF overexpression and cause B cell overactivation and immune-complex formation for HCV-associated MC syndrome development.

BAFF is a crucial factor for B-cell activation, which is associated with the anti-HCV immune response and HCV-MC development. Our results showed that HCV-induced GU-enriched miRNAs (e.g., miR-122, let-7b, and miR-206) could induce BAFF upregulation in macrophages through exosome transmission and TLR7 activation. Hepatocyte-derived exo-miR-122-induced BAFF could stimulate B cell proliferation and activation, thus producing anti-HCV antibodies during HCV infection (Fig. 6A). We observed significantly increased BAFF levels in sera from patients with HCV-associated MC syndrome, which may cause B cell overactivation, antibody overproduction, and immune-complex formation. Here, we demonstrated that the levels of BAFF expression could be regulated by HCV-induced exo-miR-122 and TLR7 SNP (rs3853839) in host cells (Fig. 6B). Hepatocyte-derived exo-miR-122 induced BAFF expression in a dose-dependent matter, suggesting that the more hepatocyte-derived exo-miR-122 was induced by HCV infection, the more BAFF was produced in macrophages. In addition, G-alleles in TLR7 SNP (rs3853839) influenced TLR7 transcription, resulting in increased BAFF expression upregulation by HCV-induced exo-miR-122. Based on our results, TLR7 SNP rs3853839 may be a potential predisposing factor in HCV-associated MC development. In the future, larger studies will be required to confirm our results.

## MATERIALS AND METHODS

### Subjects.

A total of 65 patients with HCV infection were enrolled from Taichung Veterans General Hospital (TCVGH) in Taiwan, 45 with HCV-MC and 20 chronic HCV carriers without any evidence of serum cryoglobulins or autoimmune/lymphoproliferative disorders (Table 1). HCV patients were diagnosed by using qualitative PCR to detect viral RNA (Roche, Germany) and examining the levels of serum alanine aminotransferase (ALT). The inclusion criteria for patients with rheumatic diseases met the 2002 revised version of the European criteria for Sjögren’s syndrome (SS) (49), the 2010 revised criteria of the American College of Rheumatology (ACR) for rheumatoid arthritis (RA) (50), and the 1997 revised criteria of the ACR for systemic lupus erythematosus (SLE) (51). The exclusion criteria in this study were as follows: (i) patients with positive HBsAg, (ii) subjects with a habit of alcohol consumption, (iii) subjects with concomitant malignancy, and (iv) subjects with bacterial infection within 1 week of enrollment time. Serum ALT levels and HCV viral loads were measured before and after therapy. This study was conducted in compliance with the Declaration of Helsinki and has been approved by the Institutional Review Board of TCVGH (SF16036B). The study methods were carried out in accordance with the approved guidelines, and written consent from all the participants was obtained.

**TABLE 1:** Demographic data and laboratory findings for chronic HCV patients with or without mixed cryoglobulinemia and for rheumatic patients without infection^*a*^

Variable	Mean ± SD or no. (%) of patients		
	Chronic HCV infection (*n* = 65)		Rheumatic patients without infection (*n* = 25)
	With MC (*n* = 45)	Without MC (*n* = 20)	
Age (yr) at study entry	67.2 ± 6.5	66.3 ± 5.0	62.1 ± 5.2
Gender, no. female	35 (77.8)	12 (60.0)	18 (72.0)
Disease duration (yr)	10.8 ± 5.1	8.2 ± 4.6	9.5 ± 3.2
Related rheumatic disease			
Sjögren’s syndrome	32 (71.1)	5 (38.5)	10 (40.0)
Rheumatoid arthritis	10 (22.2)	5 (38.5)	12 (48.0)
Systemic lupus erythematosus	3 (6.7)	3 (23.1)	3 (12.0)
RF (IU/ml)	56.2 ± 27.5*	46.8 ± 20.9	48.8 ± 32.5
Anti-CCP antibody, no. positive	18 (40.0)	8 (40.0)	12 (48.0)
ESR (mm/h)	29.2 ± 7.3	26.1 ± 4.2	24.1 ± 6.8
C4 (mg/dl)	17.3 ± 4.8*	35.6 ± 7.6	ND
ALT (U/liter)	56.7 ± 31.2*	35.2 ± 16.0	ND
Anti-HCV antibody, no. positive	45 (100.0)	20 (100.0)	0 (0)
HCV load at baseline (10^6^ IU/ml)	3.58 ± 3.22	3.24 ± 2.35	ND

a *, *P *<* *0.05; **, *P *<* *0.01 (between chronic HCV infection with MC versus without MC or without infection). ALT, alanine aminotransferase; C4, complement 4; CCP, cyclic citrullinated peptide; ESR, erythrocyte sedimentation rate; MC, mixed cryoglobulinemia; RF, rheumatoid factor; ND, not determined.

### Cell culture.

The PBMCs were immediately isolated from venous blood using Ficoll-Paque Premium (GE Healthcare Biosciences AB, Sweden) density gradient centrifugation. PBMCs, THP-1 cells, or Raji cells were grown in RPMI medium supplemented with 10% FBS, 1× nonessential amino acids, 100 U/ml penicillin, and 100 U/ml streptomycin in an incubator containing 5% CO_2_ at 37°C. To induce differentiation, THP-1 cells (1.0 × 10^6^ cells) were grown in media and treated with 10 ng/ml phorbol myristate acetate (Sigma-Aldrich, USA) overnight. The HEK hTLR7 stable cell line was purchased from InvivoGen (USA) and cultured in Dulbecco modified Eagle medium (DMEM) according to the manufacturer’s protocol. Huh7.5 cells were cultured in DMEM supplemented with 2% human serum, nonessential amino acids, 100 U/ml penicillin, and 100 mg/ml streptomycin at 37°C in a 5% CO_2_ incubator. For exosome study, the cells were cultured in medium supplemented with 10% exosome-depleted fetal bovine serum (Thermo Fisher Scientific, USA) after biologic treatment.

### Exosome isolation and quantification.

Samples were centrifuged at 2,500 rpm for 10 min at 4°C to remove cell debris and then filtered through a 0.22-μm filter. Serum- and Huh7.5 cell-derived exosomes were exacted by ExoQuick exosome precipitation solution (System Biosciences, USA) according to the manufacturer’s instructions. The purified exosomes were confirmed by using immunoblotting. The exosomes were quantified using a direct ELISA-based method to quantify the exosome surface marker CD63 according to the manufacturer’s instructions (System Biosciences, USA).

### Exosomal microRNA quantitative PCR.

The total exosomal miRNAs (exo-miRNAs) were extracted from the exosomes using TRIzol reagent (Thermo Fisher Scientific, USA) and purified by RNeasy MinElute Cleanup kit (Qiagen, Germany) according to the manufacturer’s instructions. A portion (25 fmol) of synthetic Caenorhabditis elegans miRNA (cel-miR-39; Thermo Fisher Scientific) was added to each sample as the internal control. The purified miRNAs were quantified at OD_260_ and OD_280_ by using an ND-1000 spectrophotometer (Thermo Fisher Scientific). The miRNA expression was quantified using a TaqMan MicroRNA assay kit (Thermo Fisher Scientific) according to the manufacturer’s protocol. Quantitative reverse transcription-PCR (QRT-PCR) reactions were performed on a StepOnePlus real-time PCR system (Thermo Fisher Scientific) using a standard protocol.

### Loading of miRNAs mimic or control into exosomes.

Electroporation was used in loading miR-122/let-7b mimic or control into Huh7.5 cell-derived exosome. In brief, 0.1 μmol of miR-122/let-7b mimic or control (Thermo Fisher Scientific) was added to 20 μl of Huh7.5 cell-derived exosome sample (approximately 5 × 10^8^ particles). The mixtures were electroporated at 500 pulse voltage/10 pulse width (ms)/3 pulse number using a Neon transfection system (Thermo Fisher Scientific). After electroporation, the mixture was immediately treated with 1 U of RNase A (Qiagen, Germany) for 30 min, followed by the addition of the 2 μl of RNase inhibitor. miR-122/let-7b mimic- or control-loaded exosomes were extracted using ExoQuick exosome precipitation solution (System Biosciences, USA) according to the manufacturer’s instructions.

### Immunoblotting.

The cells with different treatments were lysed in RIPA buffer (25 mM Tris-HCl [pH 7.6], 150 mM NaCl, 1% NP-40, 1% sodium deoxycholate, 0.1% SDS) containing a protease inhibitor cocktail (Roche, Germany). Then, 20 μg of total protein from exosome lysate was loaded onto and separated on a SDS-PAGE gel and transferred to a polyvinylidene difluoride membrane (Millipore, USA). The membranes were incubated with primary antibodies, followed by peroxidase-conjugated secondary antibodies. The results were detected using a charge-coupled device camera-based imager (GE Healthcare Life Sciences, USA) after membrane incubation with enhanced chemiluminescence (ECL) substrates (Millipore, USA).

### Genotyping of TLR7 variants.

Genomic DNA was extracted from EDTA-anticoagulated peripheral blood using spin columns with a QIAamp DNA blood minikit (Qiagen, Germany) according to the manufacturer’s instructions. The SNP of rs3853839 was genotyped by using a TaqMan SNP genotyping assay (Thermo Fisher Scientific) according to the manufacturer’s instructions.

### Statistical analysis.

The results are presented as means ± the standard deviations (SD). An unpaired, two-tailed Student *t* test was used for between-group comparisons. A one-way analysis of variance with a *post hoc* Bonferroni test was used for multiple comparisons. The correlation coefficient was calculated using Spearman’s correlation test. *P* values of <0.05 were considered statistically significant, and tests were performed using GraphPad Prism 8.

10.1128/mBio.02764-21.1 TEXT S1 Supplemental materials and methods. Download Text S1, DOCX file, 0.03 MB. Copyright © 2021 Liao et al. 2021 Liao et al. https://creativecommons.org/licenses/by/4.0/ This content is distributed under the terms of the Creative Commons Attribution 4.0 International license.
