# Supplementary material for: Hepatitis C Virus-Induced Exosomal MicroRNAs and Toll-Like Receptor 7 Polymorphism Regulate B-Cell Activating Factor
Source: mBio. 2021 Nov 2;12(6):e02764-21. doi: 10.1128/mBio.02764-21 (PMC8561394; doi:10.1128/mBio.02764-21)
Supplement: TABLE S1 [file mbio.02764-21-st001.docx]

**Table S1** Differentially expressed miRNAs in serum–derived exosomes from patients with hepatitis C virus (HCV) infection compared with those in healthy control (HC), identified by miRNA NGS analysis*.

| Up-regulated miRNAs | Fold change^§^  (median value) | Down-regulated miRNAs | Fold change^§^  (median value) |
| --- | --- | --- | --- |
| hsa-miR-122 | 3.85 | hsa-miR-1973 | 0.32 |
| hsa-miR-155 | 3.54 | hsa-miR-1275 | 0.30 |
| hsa-let-7b | 3.23 | hsa-miR-182 | 0.20 |
| hsa-miR-21 | 3.05 | hsa-miR-26a | 0.28 |
| hsa-miR-206 | 2.85 | hsa-miR-3675 | 0.28 |
| hsa-miR-193a | 2.40 | hsa-miR-151b | 0.31 |
| hsa-miR-940 | 2.38 | hsa-miR-181b | 0.20 |
| hsa-miR-200b | 2.28 | hsa-miR-876 | 0.27 |
| hsa-miR-429 | 2.25 | hsa-miR-34b | 0.24 |
| hsa-miR-374a | 2.15 | hsa-miR-190a | 0.26 |
| hsa-miR-379 | 2.12 | hsa-miR-935 | 0.32 |
| hsa-miR-1908 | 2.10 | hsa-miR-7704 | 0.25 |
| hsa-miR-4523 | 2.03 | hsa-miR-766 | 0.31 |
|  |  | hsa-miR-9-5p | 0.29 |
|  |  | hsa-miR-3687 | 0.28 |
|  |  | hsa-miR-4508 | 0.20 |
|  |  | hsa-miR-5010 | 0.23 |
|  |  | hsa-miR-6802 | 0.27 |
|  |  | hsa-miR-204 | 0.33 |
|  |  | hsa-miR-4742 | 0.26 |

*miRNA, microRNA; NGS, next generation sequencing.

^§^Fold change: if the number >2.00 or <0.33, the difference is considered significant.
