# Supplementary figures and images for: Hepatitis C Virus-Induced Exosomal MicroRNAs and Toll-Like Receptor 7 Polymorphism Regulate B-Cell Activating Factor
Source: mBio. 2021 Nov 2;12(6):e02764-21. doi: 10.1128/mBio.02764-21 (PMC8561394; doi:10.1128/mBio.02764-21)

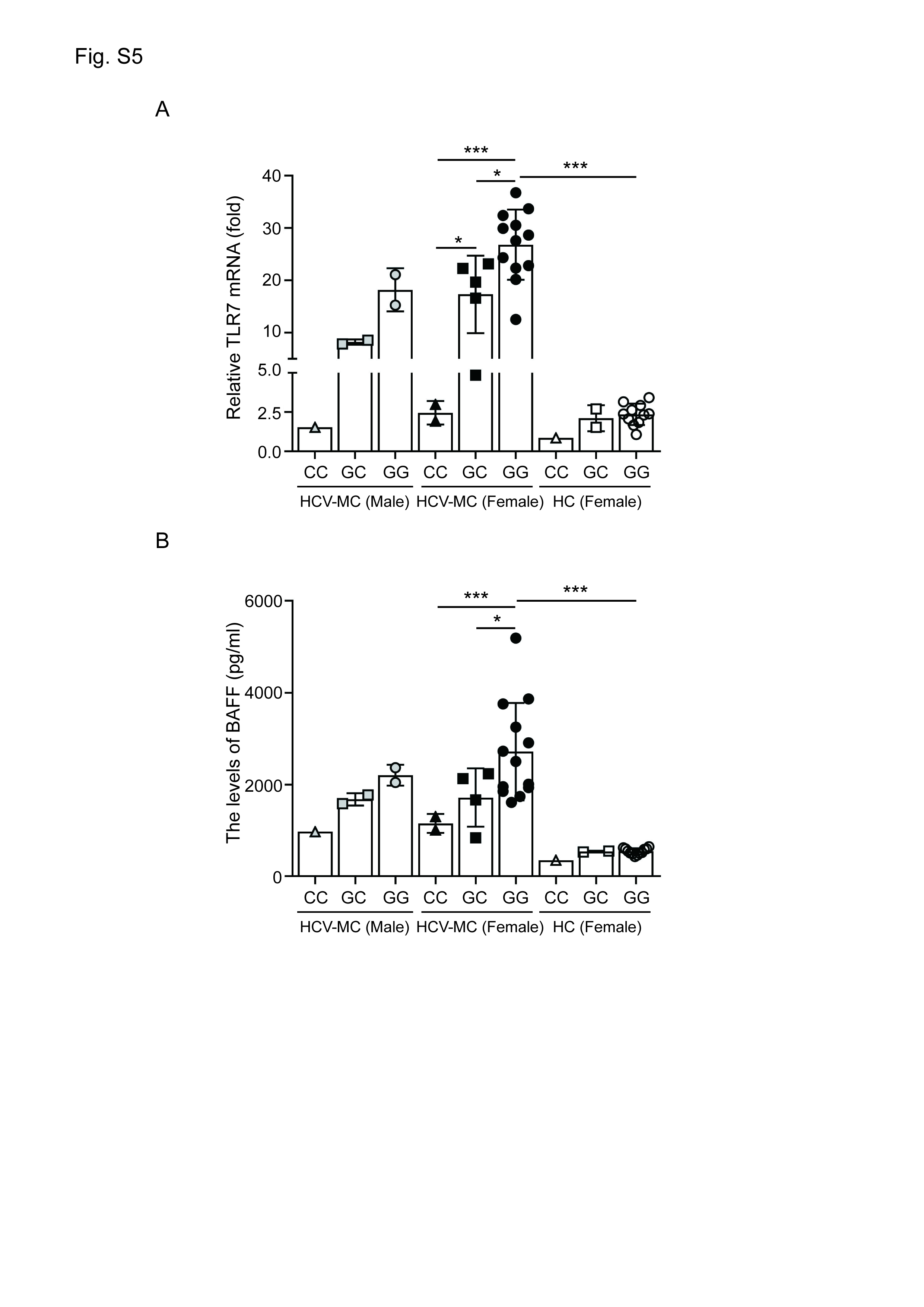

Supplement: FIG S5 [file mbio.02764-21-sf005.tif]
