# Supplementary material for: Hepatitis C Virus-Induced Exosomal MicroRNAs and Toll-Like Receptor 7 Polymorphism Regulate B-Cell Activating Factor
Source: mBio. 2021 Nov 2;12(6):e02764-21. doi: 10.1128/mBio.02764-21 (PMC8561394; doi:10.1128/mBio.02764-21)
Supplement: TEXT S1 [file mbio.02764-21-s0001.docx]

**File S1.** Supplementary Materials and Methods

***Reagent or Resource***

| Reagent or Resource | Source | Identifier (Cat No.) |
| --- | --- | --- |
| Antibodies | | |
| Mouse anti-Alix  antibody | Santa Cruz | sc-53540 |
| Mouse anti-ASGPR1/2  antibody | Santa Cruz | sc-166633 |
| Mouse anti-CD9 antibodies | Abcam | ab58989 |
| Mouse anti-CD81 antibodies | Abcam | ab79559 |
| Mouse anti-CD86 antibodies | BioLegend | 305405 |
| Mouse anti-NFκB p65 | Santa Cruz | sc-8008 |
| Mouse anti-p-NFκB p65 | Santa Cruz | sc-136548 |
| Mouse anti-TLR7  antibody | Santa Cruz | sc-57463 |
| Mouse anti-TSG101  antibody | Santa Cruz | sc-7964 |
| Rabbit anti-BAFF  antibody | Sigma-Aldrich | 07-167 |
| Rabbit anti-calnexin antibodies | Cell Signaling Technology | #2679 |
| Rabbit anti-CD63 antibodies | Abcam | ab68418 |
| Rabbit anti-TLR7 antibodies | Novus Biologicals | NBP2-24906 |
| Cell lines | | |
| HEK-Blue^TM^ hTLR7 cells | InvivoGen | hkb-htlr7 |
| Chemicals and Assay kits | | |
| CellTiter 96® AQueous One Solution Cell Proliferation Assay | Promega | G3580 |
| CpG ODN2395 | InvivoGen | tlrl-2395 |
| ExoQuick exosome precipitation solution | System Biosciences | EXOQ5A-1S |
| ExoQuick-TC | System Biosciences | EXOTC10A-1 |
| Ficoll®-Paque Premium | GE Healthcare Biosciences | GE17-5442-02 |
| Flagellin | InvivoGen | tlrl-stfla |
| Imiquimod | InvivoGen | tlrl-imqs |
| Lipopolysaccharides | Sigma-Aldrich | SI-L2880 |
| Pam3CSK4 | Invivogen | tlrl-pms |
| Phorbol myristate acetate | Sigma-Aldrich | P1585 |
| Poly (I:C) | Sigma-Aldrich | SI-P1530 |
| QIAamp DNA Blood Mini Kit | QIAGEN | 51106 |
| Resiquimod | Sigma-Aldrich | SI-SML0196 |
| RNeasy MinElute® Cleanup Kit | QIAGEN | 74204 |

| Trizol | Thermo Fisher Scientific | 15596018 |
| --- | --- | --- |
| Zymosan A | Sigma-Aldrich | SI-Z4250 |
| hsa-miR-122 miRNA mimic | Thermo Fisher Scientific | MC11012 |
| hsa-let-7b miRNA mimic | Thermo Fisher Scientific | MC11050 |
| mirVana miRNA mimic Negative Control | Thermo Fisher Scientific | 4464058 |
| hsa-miR-122 TaqMan MicroRNA Assays | Thermo Fisher Scientific | 002245 |
| hsa-let-7b TaqMan MicroRNA Assays | Thermo Fisher Scientific | 002619 |
| Human BAFF TaqMan Gene Expression Assays | Thermo Fisher Scientific | Hs00198106_m1 |
| Human TLR7 TaqMan Gene Expression Assays | Thermo Fisher Scientific | Hs00152971_m1 |
| rs3853839 TLR7 TaqMan SNP genotyping assay | Thermo Fisher Scientific | C__2259573_10 |
| Human BAFF Quantikine ELISA Kit | R&D | DBLYS0B |
| ExoELISA-ULTRA Complete Kit | System Biosciences | EXEL-ULTRA-CD63-1 |

SI Methods

***TLR ligands stimulation***

To analyze the expression of BAFF in response to innate immunity ligands, 5 × 10^5^ cells were treated with the following stimuli for 24h: Pam3CSK4 (TLR1 and TLR2 ligand, 100 ng/ml), peptidoglycan (TLR2 ligand, 10 μg/ml), poly (I:C) (TLR3 ligand, 50 μg/ml), lipopolysaccharides (LPS) from *Escherichia coli* 055:B5 (TLR4 ligand, 100 ng/ml), flagellin (TLR5 ligand, 100 ng/ml), imiquimod (R837, TLR7 ligand, 1 μg/ml), resiquimod (R848, TLR7 and TLR8 ligand, 1 μg/ml) and CpG (TLR9 ligand, 1 μg/ml). After treatment, the cells were measured using the TaqMan microRNA real-time RT-PCR Assays kit (Thermo Fisher Scientific, USA).

***MicroRNA next generation sequencing (NGS) analysis***

The small RNA library was prepared using a Total RNA-Seq kit v2.0 (Thermo Fisher Scientific, USA). Template preparation was carried out with the Ion PGM Template OT2 200 kit (Thermo Fisher Scientific, USA), according to the manufacturer’s protocol; the Ion PGM^TM^ Sequencing 200 kit (Thermo Fisher Scientific, USA) and 318 chip were used with the Ion PGM sequencer, as described in the Ion PGM^TM^ Sequencing Kit User Guide. Data alignment to the hg19 human reference genome and base calling were done using the built-in Torrent Suite software v4.0 (Thermo Fisher Scientific, USA). The differential expression analysis was carried out using Partek Genomic Suite 6.6 (Partek).

***Flow cytometry analysis for CD86 levels***

For flow cytometry analysis, cells were incubated with the PE–conjugated anti-CD86 monoclonal antibody (BioLegend, USA), and examined by flow cytometer (FACSCanto II, BD Biosciences). PE–conjugated IgG1 (BioLegend, USA) was used as an isotype control. Data were analyzed by the CellQuest software and were expressed as the mean fluorescence intensity (MFI) of CD86.
